# Real-Time Excitation of Slow Oscillations during Deep Sleep Using Acoustic Stimulation

**DOI:** 10.3390/s21155169

**Published:** 2021-07-30

**Authors:** Marek Piorecky, Vlastimil Koudelka, Vaclava Piorecka, Jan Strobl, Daniela Dudysova, Jana Koprivova

**Affiliations:** 1 National Institute of Mental Health, 250 67 Klecany, Czech Republic; marek.piorecky@fbmi.cvut.cz (M.P.); vlastimil.koudelka@nudz.cz (V.K.); jan.strobl@fbmi.cvut.cz (J.S.); daniela.dudysova@nudz.cz (D.D.); jana.koprivova@nudz.cz (J.K.); 2 Faculty of Biomedical Engineering, Czech Technical University in Prague, 272 01 Kladno, Czech Republic; 3 Third Faculty of Medicine, Charles University, Ruská 87, 100 00 Prague, Czech Republic

**Keywords:** slow-wave activity, acoustic stimulation, inter trial phase clustering, phase-locked loop

## Abstract

Slow-wave synchronous acoustic stimulation is a promising research and therapeutic tool. It is essential to clearly understand the principles of the synchronization methods, to know their performances and limitations, and, most importantly, to have a clear picture of the effect of stimulation on slow-wave activity (SWA). This paper covers the mentioned and currently missing parts of knowledge that are essential for the appropriate development of the method itself and future applications. Artificially streamed real sleep EEG data were used to quantitatively compare the two currently used real-time methods: the phase-locking loop (PLL) and the fixed-step stimulus in our own implementation. The fixed-step stimulation method was concluded to be more reliable and practically applicable compared to the PLL method. The sleep experiment with chronic insomnia patients in our sleep laboratory was analyzed in order to precisely characterize the effect of sound stimulation during deep sleep. We found that there is a significant phase synchronization of delta waves, which were shown to be the most sensitive metric of the effect of acoustic stimulation compared to commonly used averaged signal and power analyses. This finding may change the understanding of the effect and function of the SWA stimulation described in the literature.

## 1. Introduction

Sleep is a dynamic process and one of the most fundamental physical requirements for human survival [1,2]. The electroencephalography (EEG) is most used for its examination [3]. Four sleep stages are commonly detected according to the more recent guidelines published by the American Academy of Sleep Medicine (AASM) [4]: three non-rapid eye movement stages (NREM1, NREM2, and NREM3) and rapid eye movement (REM) sleep [5,6]. Deep sleep (NREM3) plays an important role in memory consolidation. NREM3 is characterized by slow wave activity (SWA) containing a frequency up to 4 Hz. Specifically, so-called slow oscillations (SOs) have a significant impact on memory [5,7,8,9,10]. SOs are synchronized EEG waves with a frequency from 0.5 Hz to 1.0 Hz [9] as a neocortical-hippocampal dialogue occurs, which allows for memory replay and redistribution into the long-term neocortical memory stores [11,12,13,14]. They predominate in deep sleep [15,16].

To enhance memory consolidation, a number of studies have been conducted to explore methods to improve SWA during sleep. Attempts to increase memory consolidation by stimulating EEG signals have used electrical, olfactory, and acoustic stimulation [17,18]. Synchronized auditory stimulation in EEG signals modulates SOs and improves consolidation of the memory [19,20]. For the right effect, it is important to stimulate the SO waves in their rising phase (upward going SO slope, going towards the up state) [21]. A number of studies are examining memory consolidation by using synchronized auditory stimulation. For example, two phase-controlled stimulation was used in studies of Ngo et al. and Besedovsky et al. [22,23,24]. The first step was an SO-negative peak detection, followed by the first auditory stimulation with individual time delay settings, and the second stimulation was 1.075 ms delayed with respect to the first one. Results from Ngo et al. [22] were compared with those of a Thalamocortical Neural Mass Model in the study of Costa et al. [25]. The study [26] compared the precision of stimulation with [22] and the author’s implementation of the Phase-locked Loop (PLL) algorithm. A method based on the PLL was used for EEG signal stimulation in studies of Papalambros et al. and Ong et al. [27,28]. Various methods are being tested for stimulation efficacy when used on different populations, such as the elderly, insomniacs, and those with psychiatric and cognitive disorders. Indeed, the optimal timing has been recently examined to reach appropriate modulation of the SOs [29]. The literature includes a comparison of different stimulation methods, mostly on healthy young volunteers. The open loop method was used in the study of Weigen et al. and involved three pulses with 1.075 s inter stimulus interval (ISI) followed by 5–9 s pause between the next three pulses [30]. This study [30] tested the acoustic stimulation on healthy young adult subjects. Auditory stimuli adjusted and targeted by an unsupervised algorithm to be phase-locked to the negative peak of slow waves single pulse were used in the case of Leminen et al.’s study on healthy young adults [31]. Debellemaniere et al. used the linear regression fitting of a sinus wave to stimulate SOs on a filtered in their study on young adults [32]. Twenty healthy young subject were tested using the PLL method with approximately 1 s ISI followed by 5–6 s pause in study of Grimaldi et al. [33]. The open loop method with 12 pulses and 1 s ISI followed by 15 s pause was used in study of Simor et al., and this study was performed on healthy young adults [34]. The closed-loop acoustic stimulation during sleep was used in study of Fattinger et al. in the case of children with epilepsy [35].

Our study investigates chronic insomnia patients. Insomnia is a sleep disorder in which individuals complain of difficulties in falling asleep, maintaining sleep or early waking from sleep last regularly for at least four weeks [36], and it is common problem in elderly people [37]. It is the most common sleep disorder; in the adult population, 30–48% have reported at least one symptom related to insomnia at some stage of their lives [38]. At the same time, insomnia has a significant impact on the quality of life. It is a significant risk factor for cardiovascular disease, hypertension, and type 2 diabetes and may lead to lower productivity at work or a higher risk of workplace accidents [36,38]. There are still many unknowns in the pathophysiology of insomnia due to its broad definition and clinical heterogeneity [38]. It is generally accepted that the pathophysiology of insomnia could be characterized by a lack of SWA. It was found by Merica et al. that the spectral power in chronic insomnia patients is lower for delta and theta band frequencies [39]. In the same study, it was found that beta band power spectral density was higher in chronic insomnia patients during the REM sleep phase [39]. A similar power spectral density was found in elderly subjects in a study by Carrier et al. [40]. The authors of that study found that, with age, there was a decrease in the power spectral density in the SWA and in the theta and sigma bands during sleep [40]. In contrast, in the beta band, power spectral density during sleep increased with age [40].

Generally, there are two acoustic stimulation methods (fixed-step and PLL-based) applied across studies that have not been quantitatively compared yet. A comprehensive explanation of both methods and their rigorous comparison is essential for the further research of acoustic stimulation. Most of the studies report results in healthy young subjects [41]. However, some studies have presented results in elderly or middle-aged subjects. Results were presented using only one implementation of the PLL method. For example, the study of Papalambros et al. presented results in elderly subjects [27], in patients with amnestic mild cognitive impairment [42] and in middle-aged adults [19,43]. One study, specificially that of Wunderlin et al. [44], shows that the effectiveness of current implementations is not yet so high that the widespread use of SWA stimulation can be considered, and this study was evaluated on the summarized results of 11 experiments. Furthermore, the target group for expanding use would be predominantly older individuals in whom it is difficult to physiologically detect continuous long-term deep sleep [45]. Therefore, it is essential to search for sensitive methods for both the detection and analysis of SWA. For this reason, we focused our research on the following points: quantitatively comparing two types of methods (fixed-step and two implementations of the PLL method) used for stimulating SWA and offering solutions for further testing.

An open question of whether induced or modulated SWA is similar to naturally occurring SOs was identified to be of high importance for clinical translation of the stimulation methods in a recent systematic review [46]. The effect of stimulation was monitored, and advanced metrics were proposed in this study on chronic insomnia patients to help elucidate the exact mechanism of stimulation.

## 2. Materials and Methods

This section describes all methods used for analysis and evaluation. It is divided into six subsections. Section 2.1 specifies the experimental design and the data acquisition. The detection method is described in Section 2.2. Two examined stimulation methods are described in Section 2.3 and Section 2.4. Section 2.5 defines the methods used for stimulation methods comparison, and Section 2.6 describes human EEG data analysis methods applied for acoustic stimulation effect quantification.

### 2.1. Experiment Design

All participants provided written informed consent before they entered the study. The study was approved by the Ethical Committee of the National Institute of Mental Health (NIMH-CZ), approval code 133/18, as a part of the study, “Acoustic stimulation during slow-wave sleep and its effects on declarative memory in insomnia.” Data were recorded using the Brainscope polysomnography system (M&I spol. s.r.o., Prague, Czech Republic) with a band-pass filter of 0.1–200.0 Hz and with a 1 kHz sampling rate. Only the EEG signals were used for consequent analysis because of focusing on acoustic neural activity stimulation.

The testing dataset is composed of 18 records from nine subjects (aged 20–52, Mean = 25.67, Sd = 10.10, 3 women) for this pilot study. Each subject underwent the stimulation and sham sleep EEG recordings (2 nights). Subjects were stimulated via pink noise during the stimulation night. The sound levels were set up individually before the recording started. Each subject determined individual sound levels in which the stimulus was sufficiently loud and not disturbing. The fixed-step method of stimulation [22] was used to identify the stimulation time after SWA detection. The sham night had the same characteristics, and the proband also slept with headphones on, but no sound was presented.

The original dataset consisted of 21 subjects, but most of the records were excluded because of small number of real detection and stimulation tags, and three records were excluded because of a problem during recording and saving recorded file. This extended dataset was used for testing purposes only with aim of making the results more reliable; see Appendix C for details.

The entire records were scored by two expert scorers. The EEG records were subsampled at 250 Hz for the subsequent analysis. The EEG records duration was 7.84 ± 0.12 h (mean ± SEM). The mean of the detected actions in real-time measurement was 142, the minimal number of detected actions was equal to 2, and the maximal number was equal to 413. The records were included into analysis if they showed 50 detections at least.

#### Simulation of Real-Time Stimulation

**Figure 1:**
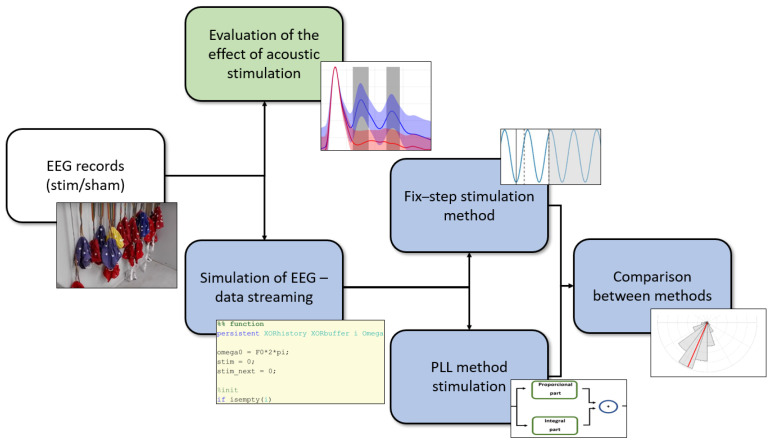
Experimental design diagram. The real EEG records were used for two separate analyses. The first branch (green) evaluates stimulation during real-time EEG records. Here, the effect of the acoustic stimulation on SWA is evaluated. The second branch (blue) shows artificially streamed data and quantitative comparison of both stimulation methods. Concrete setup and example of real recording session is attached in Appendix A.

The stimulation during real-time simulations (offline EEG data re-streaming) was performed in MATLAB software, release 2020a (The MathWorks, Inc., Natick, MA, USA), for the comparison of different stimulation methods, namely, the fixed-step stimulation [22,23] method and the PLL method [26]. The original real EEG records with a 1 kHz sampling frequency were used; see Figure 1. The easys2matlab toolbox [47] was used to import the data in MATLAB.

Uninterrupted segments of NREM2 and NREM3 EEG sleep recordings were used for subsequent analysis. These NREM stages were selected based on the expert scorers scoring of the individual phases of sleep. Only the segments with at least a duration of 5 min were analyzed. The mean duration of the deep sleep parts was 5.58 ± 0.22 h (mean ± SEM). The total numbers of detection/stimulation actions are given in Table 1 for the PPL-XOR method and for the fixed-step method.

The goal of this part was to test the fixed-step and PLL stimulation methods and to evaluate the detection and stimulation phase.

### 2.2. Detection of Slow Oscillations

**Figure 2:**
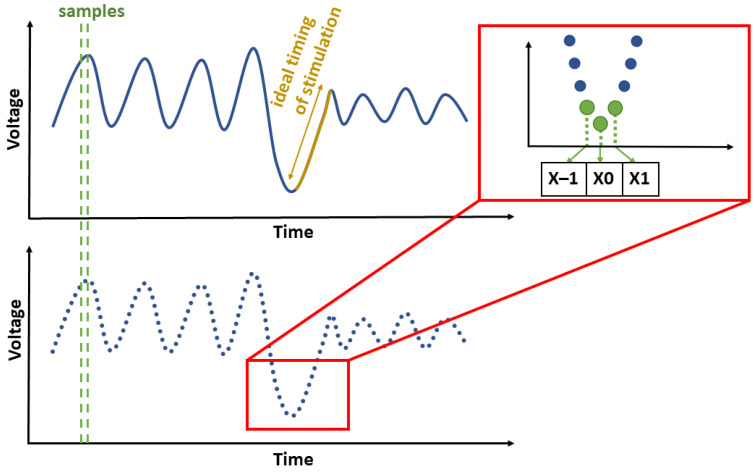
The demonstration of the slow-wave detection principle. The representative EEG signal is depicted by the blue curve. The ideal stimulation time is represented by the yellow one (the rising phase of the wave). The three following samples are taken as a reference points for minimum detection.

The slow waves need to be stimulated in their rising phase to enhance the slow oscillation rhythm [22]. The optimal detection is based on finding the ideal phase for real-time SWA stimulation; see Figure 2. The SWA minimum was detected first for this reason.

The SWA detection was applied to the reference signal. The reference signal was created by the mean from F3 and F4 EEG channels re-referenced to the mastoids (M1 and M2 electrodes). Low-pass filtering was applied to the reference signal before SWA detection. The infinite impulse response (IIR) low-pass filter (type Chebyshev, 3rd order) with a cut-off frequency of 4 Hz was used. The IIR filter was used due to the necessity for short processing times in real-time evaluation. A steeper filter was not chosen to avoid instability. SWA is characterized by a large amplitude [48]. Since an SWA minimum was detected when the negative voltage of the EEG wave exceeded −80 μV (inspired by studies [22,23]), the minimum was defined as $index(min\left(x_{-1},x_{0},x_{+1}\right))=0$.

### 2.3. Fixed-Step Stimulation Method

**Figure 3:**
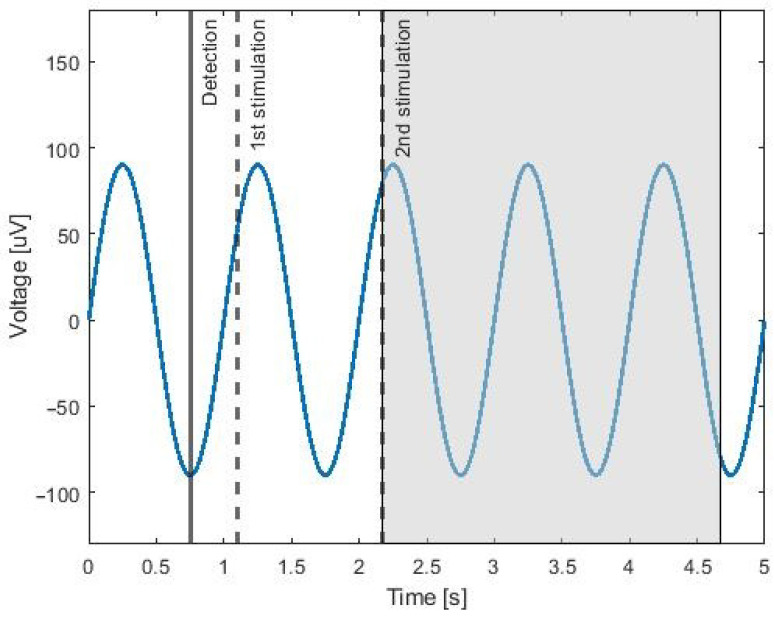
The blue curve represents the 1 Hz sine wave as an example of the EEG data. The black line represents the detection at the minimum of the signal. The dashed lines represent the 1st and 2nd stimulations. The shaded part of the graph represents the pause before the next detection.

The first tested method stimulated the SWA by a fixed time interval, which was introduced in [22,23]. The first stimulation was set to 0.350 s after the detection; see Figure 3. The second stimulation was set to 1.075 s after the first stimulation. A pause lasting 2.500 s was applied after second stimulation. All of these parameters were set based on the SWA characteristics and the fact that the stimulation should occur in the rising phase of the wave.

### 2.4. Phase-Locked Loop

Phase-locked loops (PLL) are closed-loop feedback systems consisting of both analog and digital components, including a voltage-controlled oscillator. They are used for the generation of an output signal, the frequency of which is synchronized (or locked) to that of a reference input. Phase-locked loops are used in many applications including signal generation, frequency synthesis, frequency modulation and demodulation, tone recognition, signal detection, and filtering [49].

The digital PLLs are basically designed with four components: a phase detector, loop filter, voltage-controlled oscillator (VCO), and divider [50]. The phase detector generates a signal that is sensitive to the phase difference. High-frequency components need to be suppressed by the low-pass loop filter. The VCO generates a periodic output signal [51].

More PLL types have been used in the stimulation case during sleep via EEG, as in studies [26,28]. Two types of PLL were implemented in this study. The first one was based on the exclusive-OR (XOR) principle. This PLL-XOR method was chosen for its robustness. For verifying the PLL-XOR method’s result, a second type of PLL was implemented, namely the PLL implementation with an integral part.

Both of our PLL implementations generated an artificial harmonic signal with known instantaneous frequency and phase. Stimulation was placed in a specific position in the rising phase. It was necessary to do this at the same angle in waves with different frequencies. The stimulation was performed if the PLL signal achieved a value in the predefined interval (values in which the PLL signal reaches the right phase for stimulation). This interval corresponded to 310–360${}^{\circ }$, which extends the original target phase of 340${}^{\circ }$ from the paper [27] to a 50${}^{\circ }$ interval.

#### 2.4.1. PLL-XOR Implementation

The XOR gate is one of the simplest PLL detectors [52]. This method is based on a comparison of the positive and negative values of two signals; according to this, the PLL signal is calculated. The rectangular signals are thus compared. Thus, the phase detector is independent of the amplitude of the original signal with a benefit. The XOR gate computes the output simply based on the different inputs [53], see Table 2, which represents the XOR gate implementation for our purposes.

The outputs from XOR were inserted into the buffers, and two buffers were used. The first buffer ($P_{XOR}$) contained present XOR values. The second buffer ($M_{XOR}$) contained XOR values from the recent past. Thus, the $M_{XOR}$ buffer represented the memory.

The equation representing the recomputing of the buffer’s values to the phase error can be written as follows,

(1) $$\begin{array}{c} \Delta \omega =2\pi \cdot \left(\sum^{L}P_{XOR}+k\sum^{N}M_{XOR}\right), \end{array}$$

where Δω is the frequency difference, $P_{XOR}$ is the present XOR buffer, *L* is the number of elements in the present XOR buffer, *k* is the past gain coefficient, $M_{XOR}$ is the memory XOR buffer, and *N* is the number of elements in the memory XOR buffer.

The effect of the past values is affected by the past gain coefficient together with a ratio of the present and memory buffer length. The number of elements was set to 50 in the present XOR buffer ($P_{XOR}$) and to 1000 (based on the sampling frequency of EEG records) in the memory XOR buffer ($M_{XOR}$). The past gain coefficient *k* was set to 0.1. The memory XOR buffer ($M_{XOR}$) had a twofold higher influence than the present XOR buffer ($P_{XOR}$) due to this setting. The length of the present XOR ($P_{XOR}$) determines the number of samples after which the PLL signal is going to change. The present XOR buffer ($P_{XOR}$) was set to 50 due to a quick change in the PLL signal parameters. This is a compromise between the quick change in the parameters and the number of elements in the buffer. The present angular frequency ω of the PLL signal was computed from PLL signal frequency difference Δω and the angular frequency from one time step in the past:

(2) $$\begin{array}{c} \omega =G\cdot mod\left(\omega_{past}-\Delta \omega ,2\pi \right), \end{array}$$

where mod(...,2π) is the remainder of a division by 2π. The result of this operation is multiplied by the gain coefficient *G*. The gain coefficient was set to 8 in this study because of the lower number of elements in the present XOR buffer.

#### 2.4.2. PLL Implementation with the Integral Part

**Figure 4:**
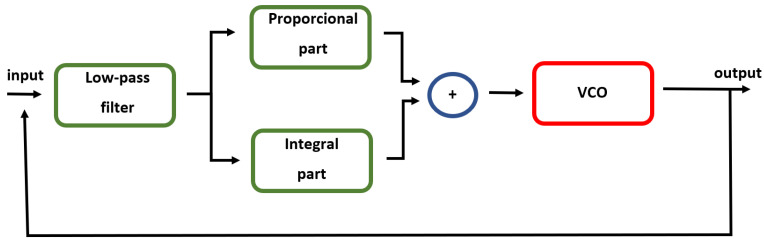
Block diagram of the PLL method used in this study.

Several implementations of the PLL method exist [50]. The first tested variant applied in this study was the basic PLL implementation using the integral and proportional parts; see Figure 4. This implementation was inspired by [51,54]. The reference EEG signal was filtered by the low-pass finite impulse response (FIR) filter first. The filtered signal $EEG_{filt}$ was multiplied by a proportional gain $G_{P}$ in the proportional part:

(3) $$\begin{array}{c} Propor=EEG_{filt}\cdot G_{P}. \end{array}$$

The integral part Integ(n) contains its previous sample Integ(n−1) and the filtered reference signal $EEG_{filt}$ multiplied by an integral gain $G_{I}$ and a sampling period $T_{s}$. Thus, the integral part Integ(n) is defined by the following equation:

(4) $$\begin{array}{c} Integ\left(n\right)=Integ\left(n-1\right)+G_{I}\cdot EEG_{filt}\cdot T_{s}. \end{array}$$

The signal error $Error_{S}$ was computed by adding both parts together:

(5) $$\begin{array}{c} Error_{S}=Propor+Integ\left(n\right). \end{array}$$

The instantaneous phase φ(n) was obtained from the signal error $Error_{S}$, the sampling period $T_{s}$, and the gain of the VCO $G_{VCO}$, which transfers the voltage quantity to a frequency quantity. It is defined by the following equation:

(6) $$\begin{array}{c} \varphi \left(n\right)=\varphi \left(n-1\right)+2\pi \cdot Error_{S}\cdot G_{VCO}\cdot T_{s}. \end{array}$$

The instantaneous amplitude Amp of the simulated PLL signal was computed simply by the sine function with the native frequency of the VCO $f_{VCO}$ and the actual time sample *t*.

(7) $$\begin{array}{c} Amp\left(n\right)=sin\left(2\pi \cdot f_{VCO}\cdot t+\varphi \left(n\right)\right). \end{array}$$

Since the PLL is rather sensitive to values of its parameters, the best fit of parameters was found through an optimization process. The whole optimization procedure can be summarized in the following steps. The training dataset was composed based on five records. The native frequency of the VCO was set such that it matched the frequency of the slow waves $f_{VCO}=0.8$. A low pass cut-off frequency $f_{c}=0.03$ Hz was set according to [26]. The gain of the VCO was set to $G_{VCO}=1$. All EEG datasets were down-sampled to Fs=100 Hz in order to make the PLL simulation faster and optimizationally feasible. Both proportional and integral part gains $G_{p}$ and $G_{I}$ were varied in the interval [$10^{-4}$,$10^{0}$]. The PLL was simulated on the training data, and each combination of varied gains was evaluated by a criterion function.

The PLL parameter values were optimized by three approaches in order to comprehensively explore the process of tuning the PLL parameters. Here, we briefly overview all criteria. The expressions for criteria enumeration are presented below.

The first criterion was calculated as a difference between the desired and instantaneous phase of the physiological signal at the time of simulated stimulation: the phase-based criterion. The second and most complex criterion was based on the time difference between the desired time of stimulation corresponding to the desired phase of the physiological signal and the time of simulated stimulation: the time-phase-based criterion. This last criterion was implemented to prevent over-fitting the PLL to the repetitive phase at lower aliasing-like frequencies. This simplest criterion was based on the same information as the fixed-step method: the fixed-time-based criterion.

The phase-based criterion can be expressed as follows:

$$mse_{PLL_{j}}=sum\left\{{\left[\phi_{EEG,desired}-\phi_{EEG}\left(t_{i,PLL_{j}}\right)\right]}^{2}\right\}/N,$$

where the $mse_{PLL,j}$ is the mean squared error between the instantaneous phase of the physiological signal at the time of simulated stimulation during the *i*-th stimulation event $\phi_{EEG}\left(t_{i,PLL_{j}}\right)$ and the desired stimulation phase $\phi_{EEG,desired}$, which was set to $\phi_{EEG,desired}=312^{\circ }$. The squared differences are summed across all stimulation events *N* across all five training subject records.

The time-phase-based criterion is stated in the following expression:

$$mse_{PLL_{j}}=sum\left\{{\left[t\left(\phi_{EEG,desired}\right)-t_{i,PLL_{j}}\right]}^{2}\right\}/N,$$

where the $t(\phi_{EEG,desired})$ is the smallest possible time corresponding to the desired phase of the physiological signal $\phi_{EEG,desired}$ simultaneously satisfying the condition $t\left(\phi_{EEG,desired}\right)<t_{i,detect}$.

Finally, the fixed-time-based fit was enumerated by

$$mse_{PLL_{j}}=sum\left[{\left(t_{i,desired}-t_{i,PLL_{j}}\right)}^{2}\right]/N,$$

where $mse_{PLL,j}$ is the mean squared error between the desired time $t_{i,desired}$ and the time of stimulation $t_{i,PLL_{j}}$ for the j-th set of the PLL parameters. The desired time was set to $t_{i,desired}=t_{i,detect}+0.35$ s, where $t_{i,detect}$ is the time of SWA detection.

### 2.5. Detection and Stimulation Evaluation Methods

The EEG records were analyzed in MATLAB software, release 2020a (The MathWorks, Inc., Natick, MA, USA). The analysis was performed partly in the Fieldtrip toolbox [55].

The first step was preprocessing. The M1 and M2 electrodes were used as a reference. The following analysis was done on an average F3/F4 signal. The data for evaluation were filtered by an IIR low-pass filter on 4.00 Hz.

The correctness of the detection and stimulation was computed via an analysis of phase at the detection and stimulation time points. This evaluation was done by Hilbert transformation [26,28].

The Hilbert transform allows one to extract a complex signal from a signal that contains only a real part. The complex signal can be represented using Euler’s formula [56]:

(8) $$\begin{array}{ccc} f_{a}\left(t\right) & = & M\cdot \exp^{i2\pi ft}, \end{array}$$

(9) $$\begin{array}{ccc} f_{a}\left(t\right) & = & M\cdot \cos\left(2\pi ft\right)+j\cdot M\cdot \sin\left(2\pi ft\right). \end{array}$$

These equations represent the analytical signal. Without any processing, EEG data have the form $M\cdot \cos\left(2\pi ft\right)$, which is an oscillatory signal that has only a real component. The Hilbert transform is an approach for extracting the imaginary part of a real-valued signal. This is done by creating and adding the phase quadrature component to the real part. The phase quadrature component is created by rotating parts of the complex Fourier spectrum of a real-valued signal [57]. The Hilbert transform does not affect the real part of the signal [56].

The phase values were visualized by the polar histograms. The polar histograms were divided into 20 bins, and the mean phase was computed and displayed. The CircStat toolbox [58] was used for the descriptive statistic evaluation of polar phases.

### 2.6. Acoustic Stimulation Effect Quantification Methods

All data analyses mentioned below (see Figure 1, green part) were performed on high-pass and down-pass filtered data 0.25–4.00 Hz provided by zero-phase finite impulse response (FIR) filters. According to the previous paper [27], the mean of the frontal electrodes (Fpz) was chosen for signal analysis. Only records (paired stim-sham) from seven subjects that contained more than 50 detections were used. Outlying data segments exceeding the amplitude limit $A_{max}=300\;\mu$V and segments with an amplitude below $A_{min}=10\;\mu$V were excluded from analyses. The length of segments was set to 7 s divided into 2 s pre-stimulus and 5 s post-stimulus intervals. It should be noted here that the final length of the segment was shortened to 4 s involving 1 s pre-stimulus and 3 s post-stimulus intervals in order to exclude boundary effects of the methods.

Firstly, the EEG signal was analyzed via a standard method found in the literature [22,23,27]. The averaged waveform was calculated from data segments that were time-locked to the onset of the first sound stimulus. The averaged waveforms corresponding to stimulation and sham conditions were statistically compared across subjects. This metric mixed both amplitude and inter-trial phase synchronization of the brain response.

Secondly, the data segments were transformed by the Hilbert transformation to obtain a signal amplitude time course. The Hilbert transform was applied to each single data segment separately. The obtained amplitude time courses were averaged across trials within each subject. The amplitude time series corresponding to stimulation and sham conditions were statistically compared across subjects. It should be noted that this step was done in order to measure the signal amplitude independently of the signal phase and the amount of inter-trial phase synchronization of the brain response.

Thirdly, the inter-trial phase clustering (ITPC) was computed to measure the inter-trial phase synchronization of the brain response independently of the response amplitude. Each data segment was Hilbert-transformed, and the obtained analytical signal was normalized to the unit-instantaneous amplitude of each time sample of each segment within each subject. The complex signal was averaged across trials, and the amplitude of the average was considered as the ITPC; see [56] for more details. The obtained ITPC time courses for each subject and condition were statistically compared.

**Figure 5:**
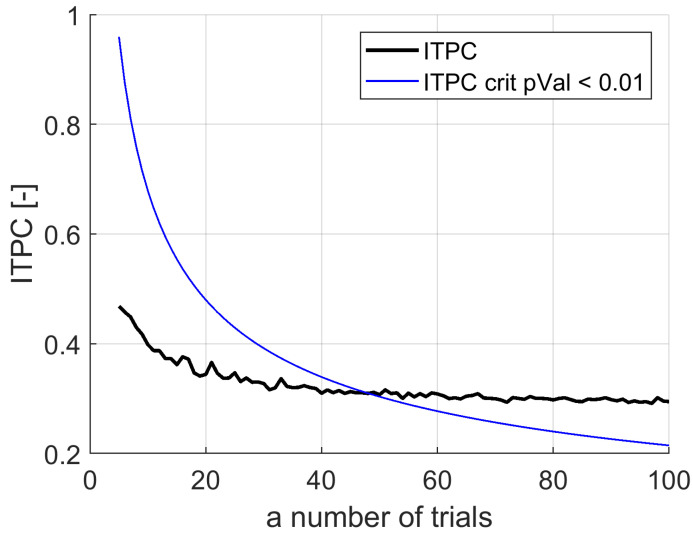
Critical ITPC values corresponding to a p=0.01 statistical threshold analytically defined by the Rayleigh Z approximation (thin blue line) and the experimentally calculated ITPC values across seven subjects (thick black line). Both phenomena were computed and printed in comparison with an increasing number of trials.

The dataset containing all night sessions and all subjects was filtered with respect to the minimum number of stimulation trials needed for significant and unbiased ITPC evaluation. Stimulation was provided by the fixed-step stimulation method described in Section 2.3. The criterion for the minimum number of trials was based on the Rayleigh Z approximation [56]:

(10) $$ITPC_{crit}=\sqrt{\frac{-ln(p)}{n}},$$

where $ITPC_{crit}$ is the critical value corresponding to a chosen *p*-value considering *n* number of trials [56]. Figure 5 depicts the computation of the mentioned criterion for the ITPC across seven subjects. A critical number of trials was set to $n_{crit}=50$, which corresponded roughly to $ITPC_{crit}=0.3$ for p=0.01.

Finally, a time–frequency representation of the brain response was calculated. The ITPC and the signal power were calculated on the frequency interval between 0.25 Hz and 25.00 Hz on the raw data segments with a length of 7 s. It should be noted that a different filter width 0.25–25.00 Hz was applied in this case compared to the previous three analyses. The short time fast Fourier transform (STFT) with the Hanning window was applied across the whole segment with a time step of 0.1 s and across all frequencies from 0.1 Hz to 25 Hz. The window length was varied from 1 s to 0.1 s corresponding to 0.1 Hz and 25 Hz, respectively.

A non-parametric statistical test equipped with cluster-based correction for multiple comparison [59] was applied to all metrics described above. The averaged waveform, amplitude, and signal power were baseline-corrected before statistical testing. The baseline interval was chosen with respect to a well-synchronized detection period from 0.5 s to 0.35 s before the first stimulus. The averaged waveform and amplitude were corrected by subtracting an averaged signal across time samples within the baseline period. The signal power was normalized to the baseline period, and the relative change in signal power in decibels was then statistically tested. The ITPC was naturally aligned across conditions due to the very precise and the same detection of the SWA in both conditions, causing the same phase synchronization during the baseline period.

## 3. Results

### 3.1. Detection/Stimulation Evaluation

**Figure 6:**
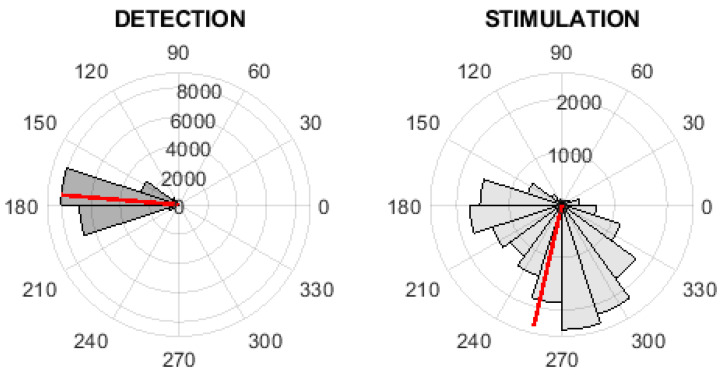
The inter-subject phase value analysis on digitally streamed real EEG records. The polar histograms of phase values at the time of detection (**left**) and at the time of first stimulation (**right**) for the fixed-step stimulation. The red line represents the mean of phase values.

**Figure 7:**
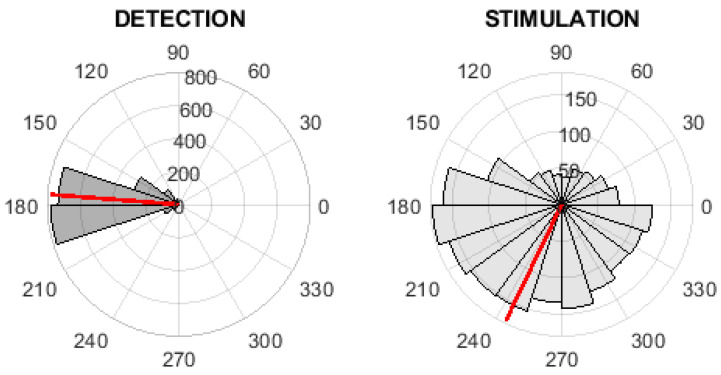
The inter-subject phase value analysis on digitally streamed real EEG records. The polar histograms of phase values at the time of detection (**left**) and at the time of first stimulation (**right**) for the PLL XOR implementation stimulation method. The red line represents the mean of phase values.

The phase values in the time of detection and stimulation were computed and visualized via polar histograms; see Figure 6 and Figure 7. These figures represent the inter-subject phase value evaluation. The red line in these polar histograms represent the mean phase value. Figure 6 represents the evaluation of the fixed-step stimulation method, where the mean value of phases is equal to 175.30${}^{\circ }$. Figure 7 represents the evaluation of the PLL-XOR method, where the mean value of phases is equal to 175.57${}^{\circ }$. The descriptive statistics parameter for the inter-subject characteristics of detection and stimulation are given in Table 3 and Table 4.

The individual characteristics of phase values in the case of detection and stimulation for both stimulation methods are included in supplementary files; see Table A1, Table A2, Table A3 and Table A4.

Given that the PLL implementation with the integral part could not be tuned, the results are summarized in the following section. The results of tuning the parameters of this PLL for all three methods are described here.

### 3.2. PLL with Integral Part Parameter Tuning

The PLL with the integral part was evaluated on the training dataset.

Different numbers of detections/stimulations were observed to set different parameters (three types of optimal parameters for three PLL tuning methods); see Table 5. The different number depends on the frequency characteristics of the PLL signal and the amplitude difference, which is the threshold for resuming detection after pacing.

**Figure 8:**
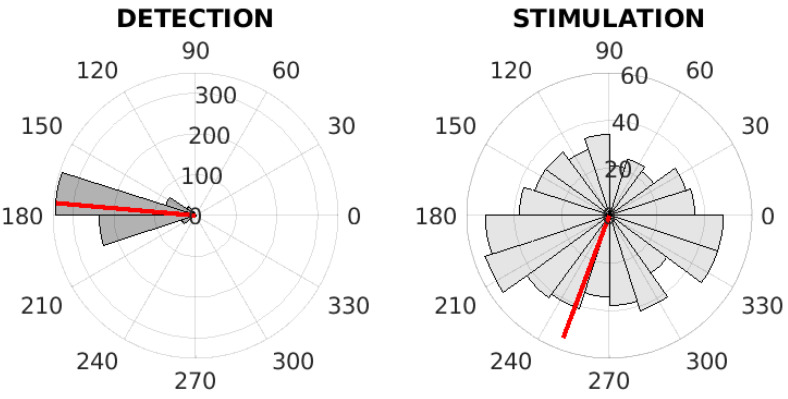
The inter-subject phase value analysis on the training dataset. The polar histograms of the phase values at the time of detection (**left**) and at the time of first stimulation (**right**) for the PLL with the integral part, the phase-based criterion. The red line represents the mean of phase values.

**Figure 9:**
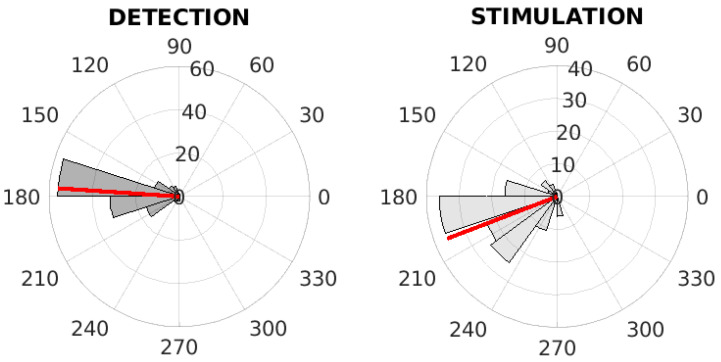
The inter-subject phase value analysis on the training dataset. The polar histograms of phase values at the time of detection (**left**) and at the time of first stimulation (**right**) for the PLL with the integral part, the time-phase-based criterion. The red line represents the mean of phase values.

**Figure 10:**
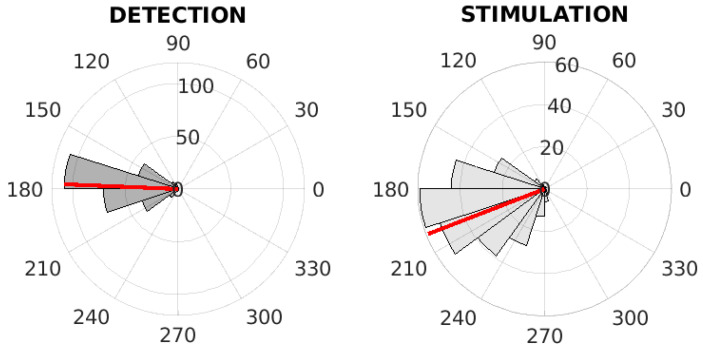
The inter-subject phase value analysis on the training dataset. The polar histograms of phase values at the time of detection (**left**) and at the time of first stimulation (**right**) for the PLL with the integral part, the fixed-time based criterion. The red line represents the mean of phase values.

Within the tuning of PLL parameters, we proposed three tuning methods, which all tuned the PLL to obtain optimal parameters. Table 6 shows the average values of the spectral range of the simulated matched PLL signal, the maximum spectral power, and the values of the most optimal parameters G1,G2. The inter-subject phase values on training dataset in case of different tuning method are depicted on Figure 8, Figure 9 and Figure 10. Please see Figure 8 for the evaluation of phase-based criterion, see Figure 9 for the evaluation of the time-phase-based criterion and see Figure 10 for the fixed-time based criterion.

### 3.3. The Effects of Acoustic Stimulation

**Figure 11:**
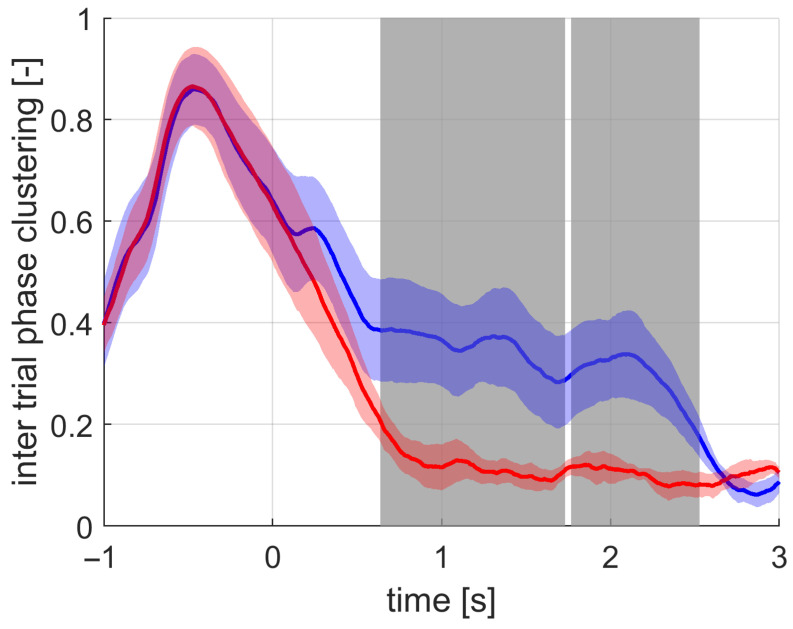
The grand average of the ITPC values across subjects for the stimulation (blue) and the sham (red) conditions. The standard deviation is depicted by the filled band around the averaged curves. The significant difference between the stimulation and sham was most pronounced at the time intervals marked with gray-filled bars.

**Figure 12:**
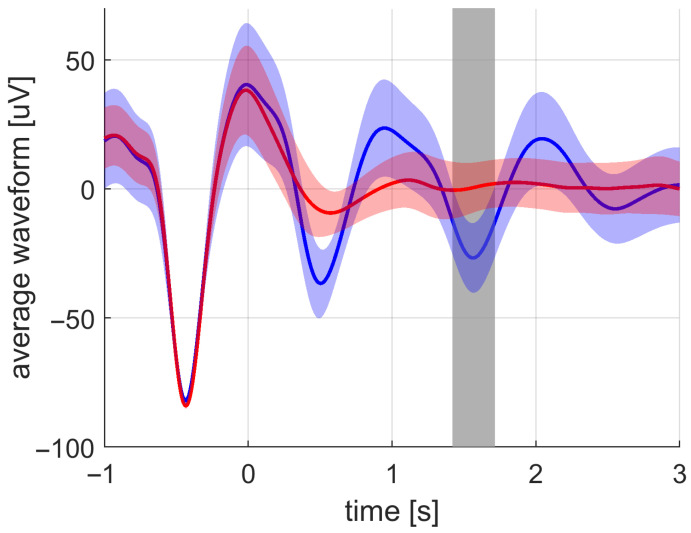
The grand average of the averaged waveform values across subjects for the stimulation (blue) and the sham (red) conditions. The standard deviation is depicted by the filled band around the averaged curves. The significant difference between the stimulation and sham was most pronounced at the time intervals marked with gray-filled bars.

**Figure 13:**
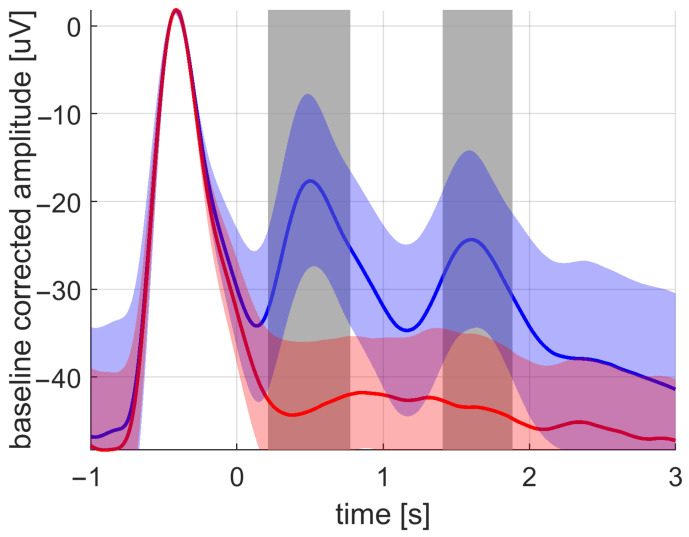
The grand average of the averaged amplitude values across subjects for the stimulation (blue) and the sham (red) conditions. The standard deviation is depicted by the filled band around the averaged curves. The significant difference between the stimulation and sham was mostly pronounced at the time intervals marked with gray-filled bars.

The EEG signal analysis revealed that the ITPC is more sensitive to changes in brain activity in a frequency band of SWA due to sound stimulus compared to the averaged waveform and amplitude metrics. The statistical increase in phase synchronization across trials lasted from 0.6 s to 2.5 s after the first stimulus; see Figure 11. The average waveform statistically increased on the interval from 1.4 s to 1.7 s; see Figure 12. The signal amplitude statistically increased on the intervals from 0.2 s to 0.7 s and from 1.4 s to 1.8 s; see Figure 13.

**Figure 14:**
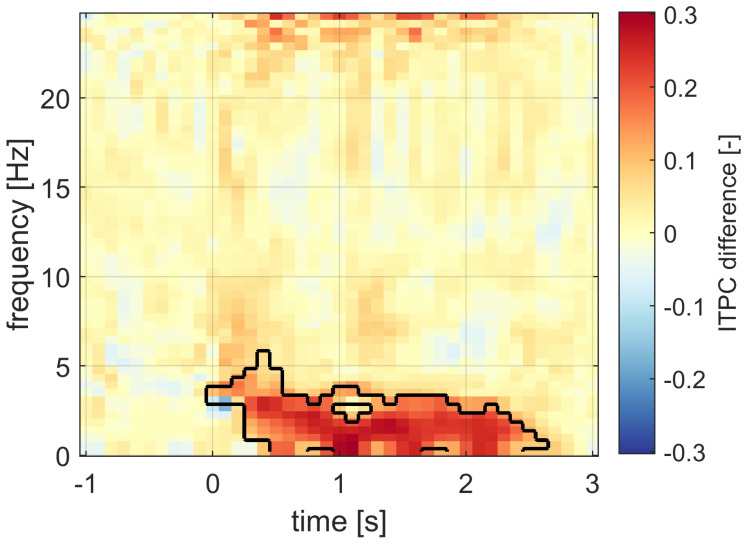
The time–frequency representation of the ITPC difference between the stimulation and sham conditions. The significant difference was most pronounced within the outlined time–frequency regions. The ITPC difference is clearly specific to the slow wave band.

The time–frequency data analysis confirmed that the robust increase in ITPC is specific to frequency band between 0.5 Hz and 4.0 Hz; see Figure 14. The stimulation effect on the phase synchronization lasted from approximately 0.2 s to 2.5 s after the stimulus application. This is also in correspondence with the ITPC time course statistics.

**Figure 15:**
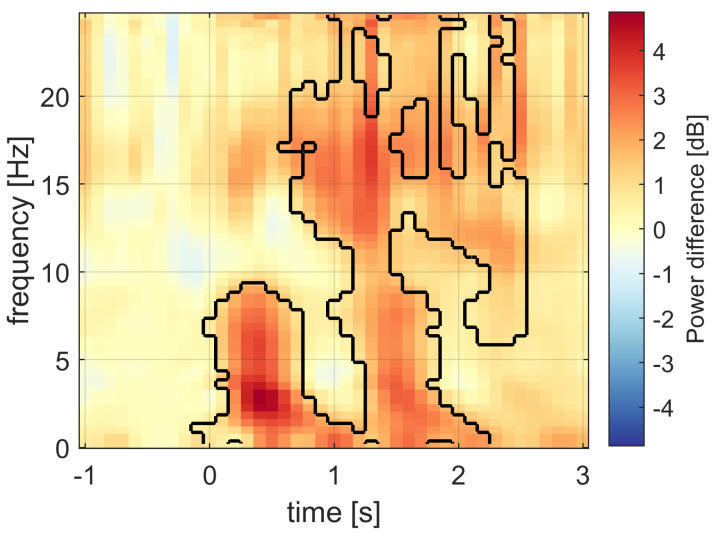
The time–frequency representation of the power relative difference in decibels between the stimulation and sham conditions. The significant difference was most pronounced within the outlined time–frequency regions. The power difference is shifted towards higher frequencies, and significant differences can be observed across multiple bands.

The time–frequency signal power representation shows that the statistically significant changes between the stimulation and sham conditions were mostly pronounced in a broadband manner; see Figure 15. The changes in power due to sound stimulation were not specific to slow waves in this case. There was an increase in delta and theta waves on the intervals from 0.0 s to 0.7 s and from 1.2 s to 1.7 s. This result confirms the finding of time-specific increases in amplitude previously mentioned and depicted in Figure 13. There was a power increase in the sleep spindle band at times from 0.7 s to 2.5 s and in the beta band during approximately the same time period.

## 4. Discussion

Research on the real-time stimulation of SWA is already being developed, and several independent scientific teams have already described their implementations and initial results. The aim of our paper was to add objectification by a quantitative comparison of the two most commonly used approaches to stimulation. Our aim was also to extend a family of acoustic stimulation effect metrics through a sensitive and well-established concept. This will help in understanding the mechanisms underlying the stimulation efficacy and basic principles, which are not yet known or clearly defined in the literature.

The first major issue of SWA stimulation is the objectification of subject measurements. Because of the real-time response, it is not possible to work with FIR filters such that they guarantee an ideal steepness, and it is generally difficult to find a metric that can ideally detect slow oscillations across different subjects [46]. There is also an issue with adjusting the sound level. In this study, the threshold was set individually so that a subject could hear it well but not be disturbed by it. For objectification, it would be appropriate to determine a threshold according to the “standardized” procedure. This may not be a large issue in younger subjects. However, in older adults, the difference in hearing quality is very high. An algorithm that would systematically determine the optimal sound level for each individual based on testing before the measurement itself is essential for future clinical studies.

SWA detection can be a problem in the case of chronic insomnia patients or, for example the elderly, due to sleep variability [60]. Insomnia is a common problem in the case of elderly people [61] as well. The NREM3 phase is not homogeneous, and fluctuating sleep occurs more frequently in these cases. In our laboratory, the course of the proband’s sleep was monitored by the constant supervision of a laboratory technician. For this reason, we switched on the detection only after the visual identification of stable deep sleep by the laboratory technicians, which is recommended for future studies.

There is a trend in the literature to replace the fixed-step method [20] with PLL methods so as to effectively stimulate the following SWA (not only the first wave after detection). There have been many implementation types of PLL, such as [26,54,62]. Two types of PLL were implemented and analyzed in this study. Specifically, PLL implementation with the integral part and PLL-XOR implementation were tested. These two types are commonly used as a digital PLL implementation.

The study [26] also implemented the PLL method with an integral part for SWA stimulation. The authors described their implementation process in detail, but we were unable to replicate some parts. For example, the cut-off frequency of the low-pass filter was set to 0.03 Hz, which was a limit that was not applicable to a standard filter in real-time processing in our case. Our IIR filters with such a cut-off frequency were unstable, and the FIR filter could not be used for real-time processing due to its slow response. For these reasons, we implemented the PLL method with an integral part based on Scher implementation [54], and the parameter set was tuned in this implementation. This implementation principally corresponded to the implementation in the study [26]. Both implementations applied a low-pass filter after the phase detector and then used an integral form to convert the filtered signal to the current phase of the PLL signal. However, the PLL method in our study also used the proportional form in the calculation. Despite small differences between our method and a previously reported method [26], both algorithms are similar enough for the the purpose of comparison.

Generally, we observed a very high sensitivity of the PLL behavior to its parameters, which ultimately convinced us to give priority to the fixed-step method. However, we approached the PLL parameter optimization in three different ways. The first approach, called the phase-based method, resulted in PLLs oscillating at very low frequencies, lower than 0.5 Hz, which was a much lower frequency band with respect to the typical SWA band between 0.5 and 4.0 Hz. It is hypothesized that the PLL fitted very slow drifts of the EEG data, which could not be attenuated by stable filters. It is not possible to apply an FIR filter for real-time stimulation due to its very high order, and the IIR filter is not stable in this case. Though the slow drifts lower than 0.5 Hz were tempered, it was not possible to completely eliminate them.

The second approach, called the time-phase-based method, was an extended form of the previous phase-based method. In that way, we eliminated the ambiguity of the phase-based method, which led to the fitting of the slow drifts. In this case, the PLL signal oscillated with a higher frequency, and the main peak in power spectral density was approximately 5 Hz. The results showed that the stimulation was performed in the rising phase of the real signal. However, the problem lay in the incorrectly high PLL frequency, causing the second stimulation to be performed in the same rising phase of the real EEG records as the first stimulation. Thus, spurious PLL fitting could be observed if the PLL frequency became very high, compared to the frequency of interest. Here, the PLL output signal frequency was approximately 5 Hz, and the frequency of the SWA was approximately 0.5 Hz.

The fixed-time-based method optimized the PLL parameters based on a prior specification of the delay between detection and stimulation. This approach was applied to avoid the influence of noise in EEG recordings, causing the noise in the phase estimates to be required by the phase-based and time-phase-based methods. Even the noise-free criterion resulted in difficulties in terms of over-fitting the PLL parameters. Very small changes in a prior fixed-time delay produced significant changes in PLL behavior, which was quantified by the mean frequency of the PLL output in our case. In this case, the PLL signal had the highest frequency across all three tested criteria, and the mean value of the phase in which the stimulation occurred did not correspond to the desired value to which the PLL was to be adjusted.

Generally, we state that the PLL method showed very complex behavior, which is not necessarily captured by the optimization metrics used in previous studies. An over-fitted PLL can result in a narrow polar histogram, while its output signal is far from optimally fitting the original EEG data. Thus, a spuriously working PLL can be obtained.

For example, it is essential to ensure that the interval in which the stimulation at the rising phase of the PLL output takes place is very narrow. Afterwards, for fast PLL oscillation, the stimulation was skipped; see Section 2.4. For this reason, there could be a small amount of stimulation events, and the PLL could therefore be wrongly fit because of the incorrectly distributed weights between subjects. To eliminate this phenomenon, we extended the stimulation interval in the rising phase of the PLL. The number of stimulations was thus increased, and the PLL signal had spectral characteristics corresponding to SWA; see Table 6. However, the resulting stimulations in the EEG were than scattered throughout the wave, including the falling phase (downward negative-going wave, going towards the down state); see Figure 8. This indicates the poor synchronization of the PLL and EEG signals.

Acoustic stimulation during slow-wave sleep can have a positive effect on memory consolidation. In recent years, many studies [17] have been published that describe the methods and the effect of stimulation in the context of memory change. However, the effect of stimulation on the electrical activity of the brain as such has not yet been clearly described. At the same time, no quantitative comparison of the two methods commonly used for acoustic stimulation was performed. The new statistical look at acoustic stimulation in our study should help others to use and develop acoustic stimulation further.

Both stimulation methods were applied on the same dataset. No brain response was elicited because the data were artificially streamed. Thus, only the first stimulation was evaluated. Overall, the fixed-step method stimulated more frequently compared to the PLL-XOR implementation method. This was due to the stimulation interval, which was too short for some fast oscillations. The fixed-time pause, which assumes the slowest frequency of 0.5 Hz, is shorter for a number of cases than the pause of the PLL method.

The fixed-step method has less variance, which could indicate greater homogeneity of the stimulation position. Shifting from the mean value of the stimuli to the rising phase would not lead to so many cases of stimulation at the falling phase. PLL-XOR has a higher value of kurtosis, which suggests that there are more extreme values in the phase distribution than in the fixed-step method. However, this difference is not significant. Skewness values are low for both methods, which indicates a relatively symmetrical distribution. A fixed-step method shows positive skewness values, while those of the PLL-XOR method are negative. We consider negative values to be advantageous here, which means that outlying values are concentrated in the left part of the distribution; i.e., stimulation occurs earlier. This means that PLL-XOR should again have the advantage that stimulation in the falling edge will not occur as often (in the case of good phasing).

The mean value of the PLL-XOR stimulation position and that of the fixed-step method are similar (approx. 250${}^{\circ }$), but the PLL-XOR method has a greater variance and a high amount of stimulation in the falling phase. Our study and comparison show that PLL cannot be easily adapted for universal use by different studied populations and individuals. When we looked at the optimal PLL parameters that were tuned for each record separately, they varied across individuals. Therefore, if we try to find unique common parameters for all individuals, we encounter the inability of PLL to adapt to our requirements. The fixed-step method does not have many cases where the falling phase of the SWA has been stimulated. The fixed-step method seems to be a better variant due to its robustness and good stimulation position results.

In this study, a combination of the ITPC and amplitude analysis was proposed to study the effects of acoustic stimulation by the fixed-step approach during sleep. In previous papers [22,23,26], the averaged signal across stimulation or sham trials was mainly utilized to demonstrate the effect of the stimulation. We showed that the ITPC is more sensitive to the effects compared to the commonly used averaged signal. The ITPC is amplitude-independent, which allows us to address the phases of the SWA specifically. This is in contrast with the commonly used averaged signal, which contains information about both amplitude and phase. For the first time, it has been shown that the phase synchronization of the SWA is increased by acoustic stimulation to a greater extent than the amplitude. An important fact is that, instead of the prolongation of SWA due to stimulation, the ITPC measures have the specific qualities of the SWA during the deep sleep period. More technically, ITPC measures how much the temporal features of the SWA are consistent across all detections. This finding can lead to a proper understanding of the actual effect of the acoustic stimulation and can give rise to more theories explaining the effects.

Further, the ITPC is the first step towards a rigorous interpretation of cross frequency coupling (CFC); see [56]. The CFC is becoming a broadly observed phenomenon in EEGs during sleep, and phase amplitude coupling (PAC) is the most common case. However, for PAC to be rigorously interpreted, the ITPC has to be known to eliminate potentially spurious couplings due to a stimulus presentation. Generally, the contribution of an evoked response to the observed changes in the SWA due to an actual sound stimulus is still an open question. We believe that computing the ITPC can contribute to a better distinction of these two mixed phenomena and will allow us to use and interpret advanced methods rigorously.

We have found that the time–frequency representations of the ITPC and signal power are not as similar as one would expect. The ITPC and signal power showed rather complementary results. The ITPC was increased in a frequency-specific band during a broad time period. The signal power changes in a more time-specific manner and are distributed over a broad frequency band. Again, the ITPC was increased mostly in the band of SWA with a longer duration compared to signal power changes.

The combination of the ITPC and power time–frequency representations is a general way of analyzing the effects of acoustic stimulation, since the ITPC is amplitude-independent and power is phase-independent. This approach is suitable for distinguishing between evoked and induced changes. For example, our results showed that the signal power in the spindle band changed due to the acoustic stimulation, while the phase synchronization did not. Thus, the spindle activation is not likely to occur due to the evoked response to the acoustic stimulus. The specific latency of the observed changes simultaneously brings further information that can be confronted with known evoked phenomena in EEGs. Utilizing the proposed approach and integrating the obtained information can shed light on the distinction between evoked and induced changes due to acoustic stimuli and can support rigorous theories explaining the treatment effects of this promising method.

## 5. Conclusions

The aim of the work was to shed light on the stimulation of SWA in sleep and its effect on spontaneous brain activity using acoustic stimuli. We were not able to achieve the optimal location of stimulation for PLL methods, since optimal parameters for PLL were not universal across individuals and the PLL behavior was too sensitive to its parameters. The fixed-step method achieved satisfactory results for chronic insomnia patients. We have concluded that the simpler approach, i.e., the fixed-step method, was more suitable for experiments in insomnia patients compared to the PLL method. It was shown that a purely phase-based quantification of the SWA resulted in the most sensitive discrimination of the stimulation and sham conditions. The ITPC and signal power are two complementary metrics that quantify the effect of acoustic stimulation. With regard to our finding that the waves are phased by the stimulation, i.e., that we do not elicit a new response in the sense of its amplitude, we only synchronize the spontaneous activity of the brain. It seems appropriate to move the stimulation closer to detection. This finding is in contrast to the previous wisdom, which is to move the stimulation time as close to the wave maximum as possible. The sooner the pulse is sent, the greater the chance of phasing the waves and thus increasing the amplitude of the average SWA. If the fixed time is shortened, the stimulation is more likely to take place at the rising phase. SWA minimum detection is simple and robust, and it is not time-consuming in real-time stimulation. Its accuracy depends primarily on the sampling frequency; in the case of 1 kHz, satisfactory performance was achieved.

## Figures and Tables

**Table 1:** Total number of actions (detection/stimulation) in the simulation case across the subjects.

	Total Number of Actions [-]		
	Minimum	Maximum	Mean
Fixed-step	255	1889	1064
PLL-XOR	278	2597	1530

**Table 2:** The XOR logical table. In this study, the first input is an incoming sample of the real EEG signal, and the second input is an incoming sample of the artificial PLL signal. Logical 1 represents the positive values of the signal, and Logical 0 represents the negative ones. The output is applied to the phase-locked loop of the PLL artificial signal.

First Input	Second Input	XOR Output
1	0	0
0	1	1
1	0	1
1	1	0

**Table 3:** Comparison of different stimulation methods in the case of the detection phase via descriptive statistics parameters. The values are in degrees except for the skewness and kurtosis coefficients.

Method	Mean	Variance	STD	SEM	Skewness [−]	Kurtosis [−]
fixed-step	175.30	2.18	15.79	0.11	0.01	0.86
PLL-XOR	175.57	3.45	19.87	0.43	0.01	0.79

**Table 4:** Comparison of different stimulation methods in the case of the stimulation phase via descriptive statistics parameters. The values are in degrees, except the skewness and kurtosis coefficients.

Method	Mean	Variance	STD	SEM	Skewness [−]	Kurtosis [−]
fixed-step	256.97	27.18	55.81	0.40	0.09	−0.03
PLL-XOR	244.29	41.48	68.94	1.50	−0.10	−0.04

**Table 5:** Comparison of the number of stimulations of the three different tuning methods in the case of PLL with the integral part.

Train Subj. ID	Phase-Based Crit.	Time-Phase-Based Crit.	Fixed-Time-Based Crit.
subj 1	52	106	86
subj 2	344	3	2
subj 3	43	13	74
subj 4	165	1	110
subj 5	134	10	9

**Table 6:** Comparison of parameters of the three different tuning methods in the case of PLL with the integral part.

Tuning Version	Spectral Range [Hz]	Max Spectrum [Hz]	G1 [−]	G2 [−]
phase-based	0.38–1.36	0.86	0.0008	0.0007
time-phase-based	4.63–4.75	4.75	0.0092	0.8555
fixed-time-based	11.82–14.68	12.48	1.0000	0.5356

**Figure A1:**
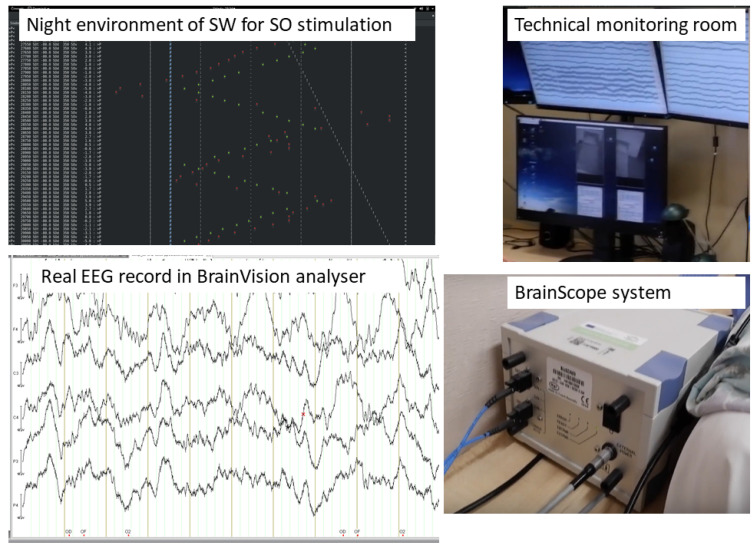
Example of the output in our SWA detection and stimulation software (**top left**), the technical monitoring room during online nightly recording of polysomnographic record used for this study (**top right**), the recorded EEG signal displayed offline in BrainVision software (**bottom left**), and the BrainScope hardware used to record the recordings analyzed in this study (**bottom right**).

**Figure A2:**
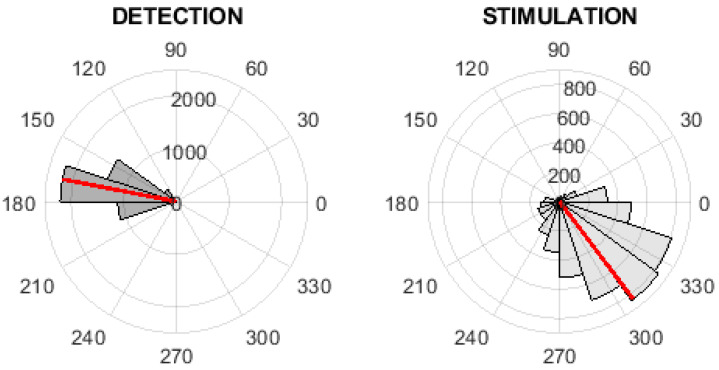
The inter-subject phase value analysis on the wilder dataset. The polar histograms of phase values at the time of detection (**left**) and at the time of first stimulation (**right**) for the fixed-step method.

## Data Availability

The data that support the findings of this study are available from the corresponding author, Vaclava Piorecka, upon reasonable request.

## Appendix B. Detection/Stimulation Evaluation

**Table A1:** Fixed-step method evaluation of detection phases via descriptive statistics parameters. The values are in degrees, except the skewness and kurtosis coefficients.

Measurement Code	Mean	Variance	STD	SEM	Skewness [−]	Kurtosis [−]
MEA01	178.68	1.10	11.25	0.26	0.00	0.93
MEA02	178.71	1.06	11.04	0.31	0.00	0.93
MEA03	177.71	1.11	11.27	0.55	0.01	0.93
MEA04	169.90	1.21	11.77	0.74	0.01	0.92
MEA05	174.21	4.03	21.48	0.52	0.02	0.77
MEA06	182.03	0.90	10.18	0.36	0.00	0.94
MEA07	170.93	2.52	16.99	0.63	0.00	0.84
MEA08	178.86	1.21	11.76	0.44	0.00	0.92
MEA09	176.07	2.06	15.37	0.43	0.01	0.87
MEA10	170.45	3.08	18.79	0.47	0.00	0.81
MEA11	177.85	1.30	12.20	0.42	0.00	0.91
MEA12	178.83	1.29	12.17	0.35	0.01	0.92
MEA13	167.92	2.14	15.66	0.43	0.00	0.86
MEA14	170.47	2.01	15.19	0.54	−0.01	0.87
MEA15	180.96	1.14	11.45	0.39	0.01	0.92
MEA16	178.66	1.56	13.39	0.44	0.01	0.90
MEA17	175.11	1.59	13.52	0.44	0.00	0.90
MEA18	169.92	3.63	20.40	0.52	−0.02	0.77
overall	175.30	2.18	15.79	0.11	0.01	0.86

**Table A2:** Fixed-step method evaluation of stimulation phases via descriptive statistics parameters. The values are in degrees, except the skewness and kurtosis coefficients.

Measurement Code	Mean	Variance	STD	SEM	Skewness [−]	Kurtosis [−]
MEA01	293.63	16.85	43.94	1.01	0.01	0.35
MEA02	287.15	17.27	44.49	1.24	0.03	0.35
MEA03	255.20	14.17	40.29	1.96	0.08	0.34
MEA04	259.04	8.53	31.27	1.96	0.03	0.55
MEA05	262.79	29.45	58.09	1.40	0.15	0.03
MEA06	312.89	16.57	43.58	1.53	0.07	0.34
MEA07	207.82	20.93	48.98	1.82	−0.21	0.24
MEA08	270.97	26.02	54.61	2.05	0.09	0.02
MEA09	279.79	31.55	60.13	1.69	0.33	0.05
MEA10	231.27	21.05	49.11	1.23	−0.01	0.09
MEA11	264.62	25.47	54.02	1.86	0.06	0.10
MEA12	273.21	10.60	34.86	1.00	0.07	0.52
MEA13	206.67	19.24	46.95	1.30	−0.27	0.34
MEA14	203.46	17.00	44.14	1.58	−0.18	0.28
MEA15	298.24	15.54	42.20	1.42	0.09	0.39
MEA16	289.72	23.08	51.43	1.70	0.13	0.18
MEA17	233.65	15.24	41.80	1.35	−0.09	0.29
MEA18	195.03	16.07	42.91	1.08	−0.23	0.34
overall	256.97	27.18	55.81	0.40	0.09	−0.03

**Table A3:** PLL-XOR implementation method evaluation of detection phases via descriptive statistics parameters. The values are in degrees, except the skewness and kurtosis coefficients.

Measurement Code	Mean	Variance	STD	SEM	Skewness [−]	Kurtosis [−]
MEA01	175.18	5.39	24.86	2.19	0.00	0.69
MEA02	180.80	1.51	13.14	1.64	0.01	0.90
MEA03	175.03	2.79	17.88	4.00	0.02	0.82
MEA04	172.66	1.20	11.73	2.62	0.01	0.92
MEA05	175.63	3.12	18.92	1.07	0.01	0.80
MEA06	184.54	0.40	6.76	0.64	0.00	0.97
MEA07	165.74	4.65	23.09	2.01	−0.01	0.71
MEA08	176.57	5.01	23.96	2.58	0.01	0.72
MEA09	179.93	0.62	8.42	0.48	0.00	0.96
MEA10	182.77	4.67	23.12	3.06	−0.01	0.71
MEA11	183,98	8.86	31.87	8.23	−0.15	0.51
MEA12	178.49	1.06	11.04	0.77	0.01	0.93
MEA13	171.37	10.30	34.36	8.59	−0.05	0.38
MEA14	166.52	3.70	20.59	2.52	0.00	0.77
MEA15	165.93	8.00	30.27	2.64	−0.02	0.54
MEA16	176.67	3.08	18.80	1.14	0.01	0.81
MEA17	160.24	4.41	22.48	2.51	0.00	0.73
MEA18	176.10	3.30	19.43	2.07	−0.01	0.79
overall	175.57	3.45	19.87	0.43	0.01	0.79

**Table A4:** PLL-XOR implementation method evaluation of stimulation phases via descriptive statistics parameters. The values are in degrees, except the skewness and kurtosis coefficients.

Measurement Code	Mean	Variance	STD	SEM	Skewness [−]	Kurtosis [−]
MEA01	224.24	39.00	66.85	5.89	−0.26	0.00
MEA02	298.87	31.93	60.49	7.56	0.07	0.05
MEA03	230.45	38.12	66.10	14.78	−0.13	−0.14
MEA04	269.53	23.18	51.54	11.52	0.13	0.19
MEA05	212.73	40.47	68.10	3.87	−0.11	0.02
MEA06	305.74	36.82	64.95	6.19	−0.13	−0.06
MEA07	200.03	25.47	54.02	4.70	−0.18	0.29
MEA08	234.40	32.78	61.29	6.61	−0.04	0.28
MEA09	319.60	36.20	64.41	3.68	−0.04	0.01
MEA10	246.41	35.22	63.53	8.41	−0.10	0.14
MEA11	221.62	31.47	60.05	15.51	−0.23	0.23
MEA12	287.88	36.62	64.78	4.56	0.01	0.03
MEA13	178.38	35.69	63.95	15.99	0.03	0.26
MEA14	202.68	18.62	46.19	5.64	−0.15	0.42
MEA15	196.56	24.91	53.43	4.67	−0.14	0.40
MEA16	268.91	40.89	68.46	4.17	−0.03	−0.05
MEA17	206.67	30.09	58.72	6.57	−0.18	0.26
MEA18	213.24	36.31	64.51	6.88	−0.22	0.11
overall	244.29	41.48	68.94	1.50	−0.10	−0.04

## Appendix C. Extended Dataset Evaluation

The whole dataset, consisting of 39 records, was used for extended analysis. The results obtained from this dataset are only supportive, as these were records with a low incidence of SWA detections. For this reason, the analysis was performed only for the fixed-step stimulation method. The aim is to support/refute the main finding of the study that the fixed-step stimulation method is sufficient. This dataset is measured with same electrode positions as mentioned in the study on insomnia patients.

The mean number of detections and stimulations was equal to 132 events. The EEG records duration was 7.92 ± 0.06 h (mean ± SEM). The deep-sleep segments’ duration was 49.58 ± 6.52 min (mean ± SEM). The minimal and maximal deep-sleep duration was 10 min and 181.32 min. Low-pass filtering was applied to the reference signal before SWA detection. The infinite impulse response (IIR) low-pass filter (type Chebyshev, 3rd order) with a cut-off frequency of 4 Hz was used.

The phase values in the time of detection and stimulation were computed and visualized via polar histograms; see Figure A2. This figures represent the inter-subject phase value evaluation. The red line in these polar histograms represents the mean phase value. Figure A2 represents the evaluation of the fixed-step stimulation method, where the mean value of detection phases is equal to 168.91${}^{\circ }$ and mean of stimulation phases is equal to 307.05${}^{\circ }$. The descriptive statistics parameter for the inter-subject characteristics of detection and stimulation are given in Table A5. These results are consistent with the assumptions and support the main outcome of our study.

**Table A5:** Descriptive statistic for case of detection and stimulation using fixed-step method of stimulation. The values are in degrees except for the skewness and kurtosis coefficients.

Type	Mean	Variance	STD	SEM	Skewness [−]	Kurtosis [−]
detection	168.91	2.29	16.20	0.23	0.00	0.85
stimulation	307.05	20.21	48.13	0.67	0.12	0.28
